# Epstein-Barr Virus (EBV)-Related Lymphoproliferative Disorders in Ataxia Telangiectasia: Does ATM Regulate EBV Life Cycle?

**DOI:** 10.3389/fimmu.2018.03060

**Published:** 2019-01-04

**Authors:** Moussab Tatfi, Olivier Hermine, Felipe Suarez

**Affiliations:** INSERM U1163/CNRS ERL8254 - Laboratory of cellular and molecular mechanisms of hematological disorders and therapeutic implications, IMAGINE Institute, Paris, France

**Keywords:** ataxia telangiectasia, Epstein Barr virus, latency, B-cell, primary immune deficiency, lymphomagenesis, hodgkin lymphoma, non-hodgkin lymphoma

## Abstract

Epstein-Barr virus (EBV) is an ubiquitous herpesvirus with a tropism for epithelial cells (where lytic replication occurs) and B-cells (where latency is maintained). EBV persists throughout life and chronic infection is asymptomatic in most individuals. However, immunocompromised patients may be unable to control EBV infection and are at increased risk of EBV-related malignancies, such as diffuse large B-cell lymphomas or Hodgkin's lymphomas. Ataxia telangiectasia (AT) is a primary immunodeficiency caused by mutations in the *ATM* gene and associated with an increased incidence of cancers, particularly EBV-associated lymphomas. However, the immune deficiency present in AT patients is often too modest to explain the increased incidence of EBV-related malignancies. The ATM defect in these patients could therefore impair the normal regulation of EBV latency in B-cells, thus promoting lymphomagenesis. This suggests that ATM plays a role in the normal regulation of EBV latency. ATM is a serine/threonine kinase involved in multiple cell functions such as DNA damage repair, cell cycle regulation, oxidative stress, and gene expression. ATM is implicated in the lytic cycle of EBV, where EBV uses the activation of DNA damage repair pathway to promote its own replication. ATM regulates the latent cycle of the EBV-related herpesvirus KSHV and MHV68. However, the contribution of ATM in the control of the latent cycle of EBV is not yet known. A better understanding of the regulation of EBV latency could be harnessed in the conception of novel therapeutic strategies in AT and more generally in all ATM deficient EBV-related malignancies.

## Introduction

Epstein-Barr virus (EBV) is a γ-herpesvirus that infects 95% of adults worldwide. EBV targets epithelial cells where lytic replication occurs, and B-cells where latent infection is established. The distinct phases of EBV infection are carefully controlled throughout the infected host's life, and chronic infection in immunocompetent individuals is mostly asymptomatic (1). Control of chronic EBV infection in immunocompromised patients may fail, leading to lymphoproliferative disorders as well as *bona fide* lymphomas (hereafter referred to as EBV-LPD) (2).

Several primary immune deficiencies (PID) are associated with poor responses to EBV and are also associated with a high risk of EBV-LPD (3). Inherited genetic abnormalities causing PID are often associated with poor or absent EBV-specific cytotoxic T-cell response and studies on PID and their underlying molecular mechanisms have led to a better understanding of immunological and cellular processes that control herpesvirus infection.

EBV infects B-cells both *in vivo* and *in vitro* and can lead to their immortalization. EBV latent genes drive the activation and differentiation of B-cells (4). Deregulation of this complex and dynamic interaction of viral gene expression and cellular activation may lead to cell transformation.

Ataxia telangiectasia (AT) is a rare PID caused by mutations in the *ATM* gene. AT patients are at increased risk of cancer, including EBV-LPD (5). However, the extent of immune compromise in AT is variable, and many patients have only minor immunological alterations (6). ATM is involved in many functions ranging from DNA repair to gene expression. Based on the paradoxical observation that EBV-LPD frequency is increased in ATM patients while they do not exhibit major T-cell deficiency, we raise the hypothesis that the defect of ATM in EBV-infected cells could play a role *per se* in the control of EBV latency, favoring a latent program more prone to lymphomagenesis. We review here the characteristics of AT and discuss the immunological and cellular abnormalities that may confer susceptibility to EBV-related malignancies.

## Clinical and Immunological Features of AT

AT is an autosomal recessive disorder caused by biallelic mutations in the *Ataxia-telangiectasia mutated* (*ATM*) gene. Its estimated incidence is about 1/300.000 live births (7). AT was first reported by Syllaba and Henner in 1926 (8), further characterized by Denise Louis-Bar (9), and finally named by Boder and Sedgwick (10).

AT is characterized by progressive neurodegeneration leading to ataxia, oculo-cutaneous telangiectasia, variable degrees of immune deficiency, and susceptibility to cancer. AT is clinically heterogeneous, the classic form starts typically around 4 years of age, most patients becoming wheelchair-bound by the age of 10. Milder forms of AT may appear later and develop slowly. AT patients have a reduced life expectancy with a median survival of 19 to 25 years (11, 12). Mortality is mostly due to respiratory tract infections and cancers (6).

AT patients have a variable immunodeficiency, rarely progressive, with some patients not affected at all. Complete loss of ATM kinase activity leads to a more severe immunologic phenotype. B-cell and T-cell lymphopenia may be present in ~70% of AT patients (6, 13). Over 60% of patients also have abnormal serum immunoglobulin levels, most notably a deficiency of IgG4 (65%), IgA (63%), and IgG2 (48%) (6).

## Genetic and Molecular Basis of AT

The *ATM* gene (~160kB) was cloned in 1995 (14). Over 400 mutations of *ATM* have been reported, spanning all 66 exons of the gene (Leiden Open Variation database). Most of these mutations lead to complete loss of ATM protein expression, but missense, and splice mutations can lead to the expression of a protein with residual kinase activity (15).

**Figure 1:**
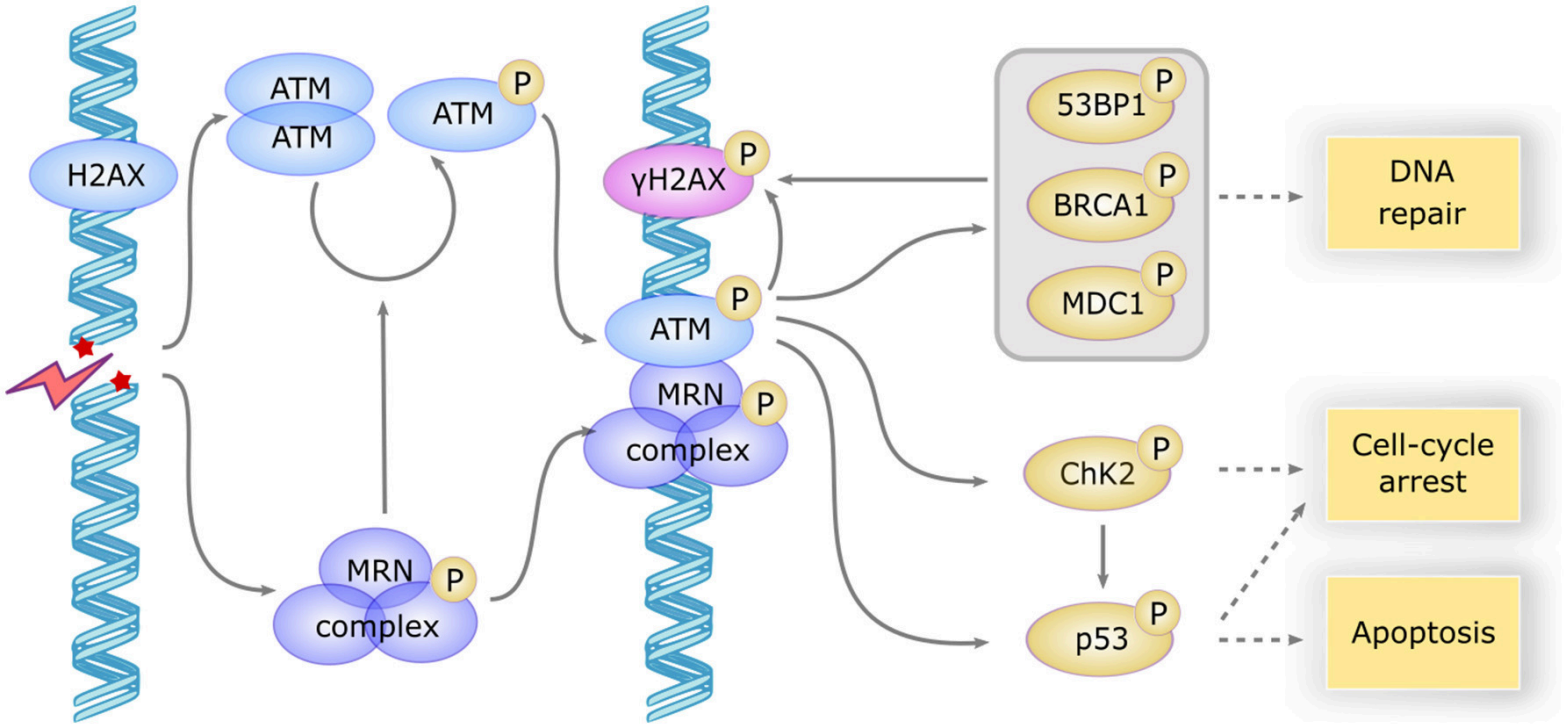
ATM activation and downstream signaling in response to DSB. DSB induces a rapid activation of the ATM dimer and the MRN complex, which in turn induces the autophosphorylation and monomerization of ATM protein. ATM becomes fully active and phosphorylates a large subset of downstream proteins including γH2AX which serves as a scaffold for the recruitment of repair proteins, MDC1, BP53, and BRCA1 involved in DNA repair, CHK2 involved in cell cycle arrest and p53 involved apoptosis induction. CHK2 also phosphorylates P53 to promote cell-cycle arrest or apoptosis.

The *ATM* gene encodes a 350 kDa serine/threonine kinase belonging to the phosphatidylinositol 3-kinase-related kinase (PIKK) family (16). ATM is mostly located in the nucleus, but ~20% are found in the cytoplasm, mainly in peroxisomes, endosomes, and as soluble proteins (17). ATM is involved in many cellular functions, including cell cycle checkpoint, apoptosis, oxidative stress, mitochondrial metabolism, gene regulation, and telomeres maintenance, but one of its major roles is its involvement in double strand break (DSB) repair (18). DSB can occur by endogenous processes, during replication fork collapse, V-(D)-J recombination or class switching, and can be induced by exogenous factors such as chemotherapy. In the canonical pathway, ATM is partially activated few seconds after DSB, probably after the relaxation of chromatin adjacent to the break. The MRN complex (Mre11/Rad50/Nbs1) recognizes the site of DNA break and in turn recruits ATM (Figure 1). Autophosphorylation of the ATM dimer occurs after its association with the MRN complex and precedes the formation of the fully active monomer forms (17).

ATM then phosphorylates H2AX, a variant of the histone H2A family, forming γH2AX foci that serve as a scaffold for the recruitment of DNA repair proteins such as MDC1, 53BP1, and BRCA1. Several other important partners are phosphorylated by ATM, such as ChK2 and p53, which initiate the downstream events of DNA repair and induce cell cycle arrest or apoptosis if DNA repair fails (18). The repair mechanism involves non-homologous end joining, an error-prone process occurring in G1/S phase (19), or homologous recombination, a faithful process in G2/M when sister chromatid is available (20).

As a result, defective DSB repair in AT patients elicit genomic instability that leads to B-cell and T-cell lymphopenia, premature senescence, and cancer. ATM plays a key role in the development of lymphocytes, allowing DSB repair occurring during B-cell, and T-cell differentiation. However, DSB repair may be possible via the alternative end-joining, an error prone, and poorly understood ATM independent pathway, which could explain the modest degree of lymphopenia in AT patients (21). Some lymphoid malignancies, such as mantle cell lymphoma, diffuse large B-cell lymphomas, and Hodgkin lymphomas, are also associated with acquired ATM mutations (22).

Patients with AT are at increased risk of cancer, especially lymphoid malignancies (23). To estimate precisely the risk of cancers in patients with AT, we conducted a retrospective study of cancers of 279 AT patients from the registry of the French National Reference Center for Primary Immune Deficiencies (CEREDIH) (5) and found that 25% of AT patients develop malignancies, the most common of which were aggressive non-Hodgkin's lymphomas (55% of all cancers), followed by Hodgkin's lymphoma (17%), leukemia (16%), and various solid tumors (12%). EBV was associated with 100% of Hodgkin's lymphomas and 50% of B-cell non-Hodgkin's lymphomas.

## Epstein-Barr Virus

EBV belongs to the *herpesviridae* family of large enveloped double-stranded DNA viruses, and was first identified in Burkitt's lymphoma in the 1960s (24). All herpeviruses have 2 distinct phases of infection, lytic, and latent. The lytic EBV infection occurs in oropharyngeal epithelial cells and leads to viral replication and production of multiple virions that spread into the underlying lymphoid tissues and infect naive B-cells (25). In the latter, EBV enters latency, a phase during which the viral genome is maintained as a nuclear episome, and only a few viral genes are transcribed (26).

Expression of the latency genes is tightly controlled by cellular and viral factors. Cellular immunity is also strongly induced during EBV infection and contributes to the elimination of infected cells expressing immunogenic lytic and latent antigens (27). Analysis of viral gene expression in EBV-associated cancers has led to a model in which EBV transitions through several latency gene expression programs (latency III, II, I) (28). Depending on the stage of latency, cells can express nuclear proteins (EBNA-1,−2,−3a,−3b,−3c, and -LP), latent membrane proteins (LMP-1,−2A, and−2B), non-coding RNAs (EBER-1 and−2), and several microRNAs (4). According to this model, EBV uses different latency programs to exploit B cell maturations pathways in the germinal center, leading the infected B-cell to the long-lived memory B-cell pool (28). The viral proteins LMP1 and LMP2 replicate the signals induced in B cells during the germinal center reaction and cause their proliferation. LMP1 mimics the signal of the activated CD40 (29) and induces EBV-LPD in transgenic mice (30), and LMP2 mimics the signal of the antigen activated B-cell receptor (31) and allow B-cell development even in the absence of normal B-cell receptor signaling (32).

EBV latency genes and non-coding RNAs may have oncogenic properties or interfere with cellular activation pathways leading to proliferation of B-cells as immunoblasts (33). Indeed, *in vitro* EBV infection of naïve B-cells leads to immortalized and fully transformed lymphoblastoïd cell lines in latency III that can induce tumors in nude mice (34). During latency III, EBNA-2 interacts with the target of the Notch pathway RBP-Jκ (35) and recruits co-activators, allowing the transcription of many genes involved in proliferation including *MYC* (36). EBNA-2 also promotes the transcription of all other latency genes, including the EBNA-3 family which gradually replaces EBNA-2 in its association with RBP-Jκ (37). The EBNA-3 family recruits co-repressors and inhibits the transcription of EBNA-2 induced genes. Indeed, prolonged expression of MYC could induce early senescence and be harmful to the virus (38). Infected B-cells migrate to the germinal center and progressively lose the expression of EBNA-2 and EBNA-3, escaping the immune control (latency II). During their transit through the germinal center to finally reach the memory B-cell pool, infected B-cells further restrict the expression of latency genes to express only EBNA1, a viral protein involved in the tethering of the viral episome to the nuclear chromatin (latency I) (39). EBV persists then indefinitely in memory B-cells (40). When these infected cells differentiate into plasma cell following an encounter with their cognate antigen, the transcription factor XBP-1 induced during plasma cell differentiation activates the expression of BZLF1, a viral protein sufficient to induce the lytic cycle (41). Virions are then released into the bloodstream and can re-infect the oropharynx epithelium to perpetuate the infectious cycle.

Occasional lytic replication occurs in the oropharynx of healthy individuals, and evidence shows that the majority of these cells do not complete the full lytic cycle despite BZLF1 expression (42–44). Abortive cycles have also been described in EBV-LPD such as Burkitt lymphomas (45), or diffuse large B-cell lymphomas (46). Several BZLF1-induced viral genes have anti-apoptotic or immunomodulatory properties, allowing the lytic cell to avoid cell-death (47). These genes are also activated during the abortive cycle and growing evidences suggests that these genes may contribute to lymphomagenesis. Indeed, the rate of EBV-LPD induced by EBV infection of humanized mice is severely reduced when BZLF1-deficient virus is used (48).

## Mechanisms Underlying AT Susceptibility to EBV

### AT Immune Dysfunction

Cytotoxic T-cells (CTL) play a major role in controlling the expansion of EBV infected B-cells. Primary EBV infection in young adults leads to infectious mononucleosis associated with a massive CTL expansion (49). The viral EBNA-3 proteins and, to a lesser extent, EBNA-2 induce a potent CTL response (27), which eliminates most infected cells.

Thymic hypoplasia has been described in AT (13), and may be the cause of the various degree of T-cell lymphopenia, especially of the naïve T-cell population (CD3+ CD4+ CD45RA+ and CD3+ CD8+ CD45RA+), found in these patients (50). Similarly, TCR excision circles (TRECs) as a measure of thymic output, can be useful for early diagnosis of AT (51). The naïve T-cell defect may also contribute to the described defect in IFNγ (52) which is important for defense against viruses and bacteria, and immunosurveillance of cancers.

However, severe viral or opportunistic infections are not frequent (6) and most AT patients seem to have an intact T-cell response (53, 54). The vaccine response is also functional, with a totally normal response for some patients and a reduced response for others (6).

Nonetheless, AT patients have recurrent sinopulmonary bacterial infections that seems to increase with age (55). This could be explained by the IgA deficiency associated with an increased risk of chronic rhinosinusitis (56), the IFNγ production deficiency (52), but also by the progressive neurodegeneration; AT patients may have mastication and swallowing difficulties that worsen with age leading to an unintentional inhalation of food (6, 57).

Several observations suggest that γδ T-cells play a role in the control of viral infections. γδ T-cells represent 1–10% of the total T-cells and recognize a distinct range of antigenic targets (58). Infusion of pamidronate (known to activate γδ T-cells) in humanized mice significantly reduced EBV-LPD. AT patients seem to have an increase in the γδ T-cell population (59).

A humoral response is also generated but plays a limited role in the control of EBV infection (60). The NK-cells response is also important in the control of primary infection (61) but the number of NK-cells and their function in AT patients seems normal (62), despite an expansion of the CD56 bright population (CD3- CD16+ CD56+). This population is important for cytokines production but is not sufficient to overcome the IFNγ production defect (52). In summary, these immunological defects in AT patients do not seem sufficient to explain the increased incidence of EBV-LPD.

Some studies have also suggested a role for invariant NK-T cells (iNKT) in EBV control. These cells are restricted by CD1d, a class I MHC-like molecule exposing lipid antigens. Patients with mutations in *SH2D1A* (encoding SAP) or in *BIRC4* (encoding XIAP) have little or no iNKT and are very sensitive to EBV. However, these mutations also affect normal T-cells function, making it unclear if the iNKT defect is responsible for the disease (63, 64). Infused iNKT in immunodeficient mice injected with EBV transformed cells show reduced tumor formation (65). Similarly, a study on EBV-infected peripheral blood mononuclear cells *in vitro* showed higher transformation efficiency when iNKT were previously depleted (66). There has been to date no full exploration of iNKT levels in AT patients, but a small study of 3 patients suggests that AT patients do have iNKT deficiency (67).

While most PID patients with EBV sensitivity have an anti-EBV CTL defect, other PID patients have a specific EBV sensitivity by other mechanisms, such as XMEN (mutations in *MAGT1)* (68), or patients with mutations in *CTPS1* (69). MAGT1 allows a TCR-induced influx of magnesium that activates T-cells (70) and CTPS1 allows CTP synthesis involved in nucleic acids anabolism (69). T-cells from these patients can respond to a standard stimulation of the immune system, but the ability of their T-cells to cope with the overwhelming proliferative stress induced by EBV infection is severely impaired, leading to an EBV specific immune deficiency.

### Evidence for a Role of ATM During EBV Infection

As mentioned above there is evidence pointing to an abnormal control of EBV infection in AT patients without unequivocal evidence for an associated cellular immune defect leading to EBV-LPD. In the face of this apparent paradox, a cell intrinsic defect leading to impaired control of EBV latency in B-cells from AT patients, thereby promoting the oncogenic properties of the virus, may be hypothesized. There is indeed some evidence demonstrating the implication of ATM in the lytic and latent cycle of EBV as discussed below.

#### During the Lytic Cycle

**Figure 2:**
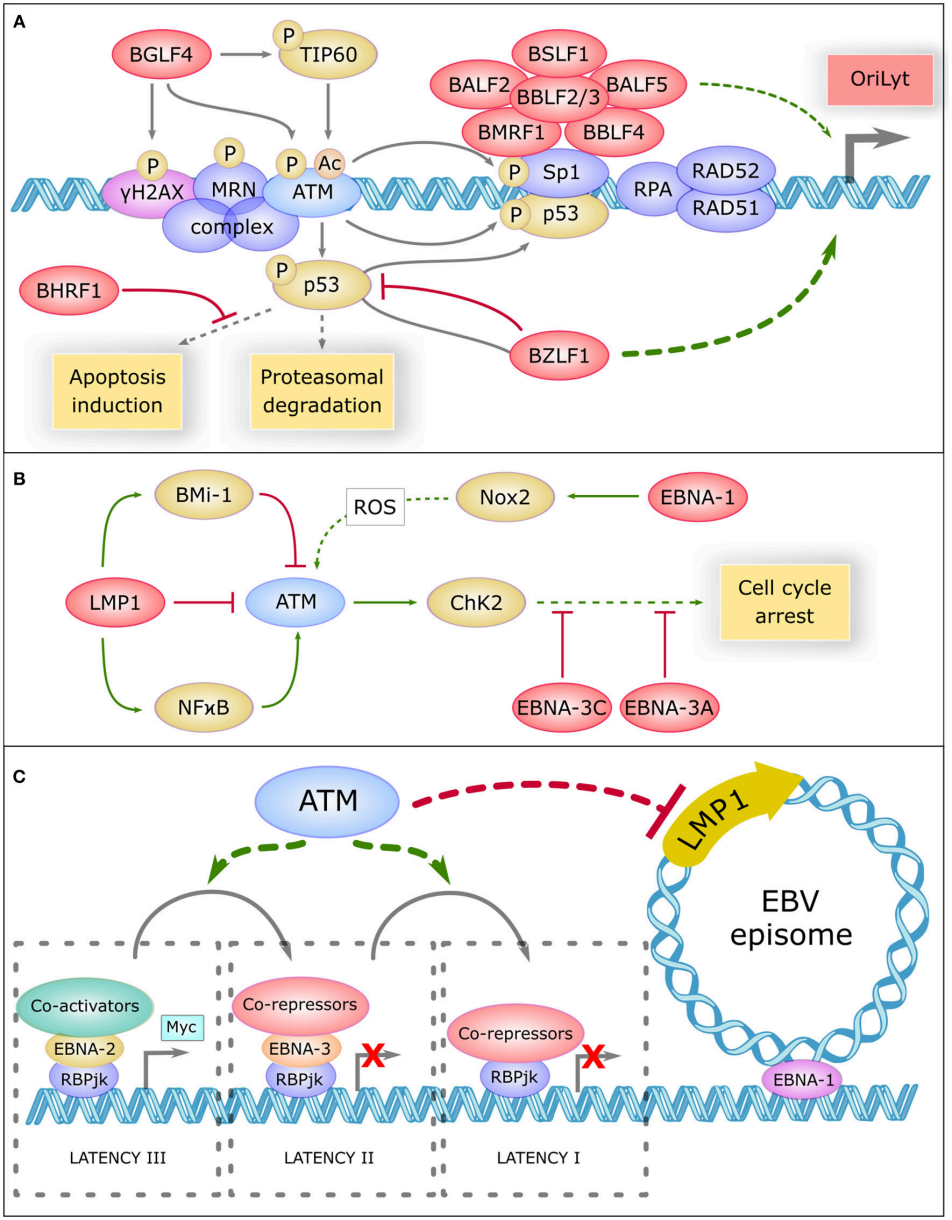
Role Of Atm In Ebv Life Cycle Regulation **(A)** The central role of ATM in the replication compartment of EBV. In the lytic cycle, DNA damage response proteins such as γH2AX, the MRN complex, ATM, SP1, RPA, RAD51, and RAD52 bind the viral genome and promote replication of the virus. Viral proteins are shown in red. BGLF4 phosphorylates H2AX, ATM, and TIP60 which acetylate ATM to promote this replication. ATM phosphorylates and activates Sp1 which is necessary to the formation of the replication compartment comprising a large complex of six core viral replication proteins (BSLF1, BALF2, BBLF2/3, BALF5, BMRF1, and BBLF4). ATM phosphorylates and activates P53, which is inhibited and driven by BZLF1 to the replication compartment. BZLF1 is a major transactivator of the lytic genes promoter OryLyt. P53 binds to Sp1 and promote the activation of OryLyt. P53 is regulated by proteasomal degradation and can induce apoptosis, but BHRF1 inhibits a panel of pro-apoptotic proteins. **(B)** ATM is regulated by EBV during latency. In the latent cycle, LMP1 downregulates ATM, and upregulates Bmi-1 which also downregulates ATM. On the other hand, LMP1 activates the NFκB pathway which activates ATM. EBNA-1 upregulates NOX2, which generates reactive oxygen species (ROS) that could activate ATM. Once activated, ATM activates CHK2 which promotes cell cycle arrest. However, EBNA-3C and EBNA-3A inhibit many proteins involved in cell cycle control. **(C)** Potential involvement of ATM in the regulation of EBV latency. ATM could be involv ed in inhibiting the expression of certain viral oncogenes, such as the main viral oncogene LMP1. ATM could also favor the progressive restriction of EBV latency, from type III latency to type I. In type III latency, EBNA-2 interacts with the target of the Notch pathway RBP-JK, recruits coactivators and induces the transcription of pro-proliferative genes like Myc. In type II latency, EBNA-3 replaces EBNA-2, and recruits co-repressors, thus preventing prolonged expression of MYC. In type I latency, RBP-JK is associated with corepressors and only EBNA-1 remains expressed, which allows the attachment of EBV episome on cellular chromosomes.

ATM operates in the regulation of the lytic cycle of many viruses including EBV. During this cycle, viral replication generates a large amount of double-stranded linear DNA in the nucleus that are recognized as double strand breaks and thus activate the repair machinery (71). ATM and the MRN complex have been shown to bind the viral genome and recruit other proteins such as RPA, RAD51, and RAD52 that promote replication of the virus. Recent studies have reported inhibition of viral replication after pharmacological inhibition of ATM (72). BGLF4, one of the first viral proteins expressed during the lytic cycle, directly phosphorylates ATM, and H2AX (73). BGLF4 also phosphorylates and activates TIP60 (74), a histone acetyltransferase, which in turn activates ATM (75) (Figure 2A).

During lytic replication, ATM activation allows the phosphorylation of P53 and SP1, a transcription factor involved in DNA repair (76). SP1 plays a role in the formation of the nuclear replication compartment of the virus (72) where a high level of P53 is found. SP1 and P53 form a complex which binds and activates BZLF1 promoter (71), the major viral transactivator of the EBV lytic genes. Other repair proteins present in these compartments such as RPA, RAD51, and RAD52 also appear to be involved in the induction of BZLF1 because their knockdown greatly reduces viral replication (77). In addition, the activity of CyclinA/CDK2 and cyclinE/CDK2 complexes appears enhanced in this context, leading to a prolonged pseudo-S phase environment that promotes replication of the viral DNA (71).

EBV uses ATM activation to facilitate its own replication. But long-lasting activated ATM may promote P53 accumulation and apoptosis induction. During the lytic cycle, the level of P53 is constant despite recurrent activation of the DNA repair pathway, and appears to be regulated by proteasomal degradation (71). In addition, BZLF1 associates with P53, inhibits its transactivating activity and drives it to the EBV replication compartment (71), which greatly limits the ability of P53 to activate pro-apoptotic genes. Even in that case, BHRF1, a viral analog of the BCL-2 protein expressed early during the lytic cycle, inhibits a large panel of pro-apoptotic proteins such as BIM, BID, BAK, or PUMA (78).

#### During Latency

ATM is also involved in the early steps of EBV latency establishment where it plays a tumor suppressor role. *In vitro*, the early hyperproliferation period of infected B cells is associated with ATM activation, leading to the death of the majority of cells (79). A total of about 3% of infected B-cells survive and become indefinitely proliferating lymphoblasts (80). Some EBV latency proteins have been shown to interact with ATM as well as with other DNA damage related proteins, but the overall implication of ATM in the latent cycle remains to be explored.

LMP1 upregulate BMI-1 in Hodgkin's lymphomas, a Polycomb related protein, and both proteins combine their effects to downregulate ATM expression (81). Similarly, EBV infection of the EBV negative BJAB line showed a defective DNA damage response (82). In addition, biopsies of patients with EBV-positive nasopharyngeal carcinoma (NPC) revealed downregulation of ATM protein levels (83). On the other hand, it has been reported that LMP1 positively regulates ATM in NPC by activating NF-κB pathway (84). This divergence in the effect of LMP1 on ATM expression is unclear and may be due to different LMP1 expression levels or to the use of different cell line types (Figure 2B).

EBNA1 upregulates the catalytic subunit of Nox2 in the NADPH oxidase complex, inducing the production of reactive oxygen species that could activate ATM (82). The EBV-infected BJAB cells expressing EBNA1 also show more chromosomal aberrations (82). EBNA3C, a viral protein essential for transformation, has been shown to attenuate DNA damage response pathways in the early steps of transformation. It also inhibits the activity of many proteins involved in cell cycle control, such as P14, P16, P27, CHK2, P53, BUBR1 (85–90). EBNA3A appears to collaborate with EBNA3C in the inhibition of P14 and P16 (85).

There is also evidence pointing to a role of ATM in the regulation of latency of Kaposi's sarcoma associated herpesvirus (KSHV) and Murine γ-herpesvirus 68 (MHV68), two herpesviruses closely related to EBV. During KSHV latency, there is a steady phosphorylation of a small amount of ATM and γH2AX, which play a role in LANA-1 transactivation, the major latency protein (91). During MHV68 latency, ATM plays a role in the transactivation of the LANA-1 analog protein ORF73. Inactivation of ATM significantly reduces the expression of LANA-1 (91) and ORF73 (92), respectively, demonstrating the importance of ATM in the control of KSHV and MHV68 latency.

## Discussion

The increased incidence of malignancies in AT has primarily been linked to the genetic instability caused by DNA repair abnormalities. The high rate of association of B-cell malignancies with EBV may be interpreted as the consequence of AT associated immunodeficiency. Most patients however do not present opportunistic infections, indicating the absence of profound cellular immune deficiency. Although data are scarce, AT patients appear to have iNKT deficiency which may participate to the lack of control of viral infections. The contribution of iNKT deficiency in the propensity of AT patients to develop EBV-LPD requires further study.

Beside immune deficiency, ATM could also contribute to AT-associated EBV-related lymphoid malignancies by interfering with the B-cell intrinsic regulation of EBV persistence. ATM is known to play a role during the lytic cycle of EBV by creating the replication compartment of the virus and by promoting its replication. During latency, several viral proteins appear to interfere with ATM expression or with its downstream signaling. However, the effect of ATM on the regulation of viral latency is not yet known. The fact that ATM plays a role in the regulation of latency proteins of EBV-related herpesvirus, such as KSHV or MHV68, suggests that ATM may also be involved in the control of EBV latency.

Recent studies have shown an involvement of ATM in the inhibition of gene expression. Indeed, ATM activation in the vicinity of the DSB promotes the ubiquitination of nearby H2A histones. This prevents the progress of polymerase II and thus inhibits the transcription of nearby genes (93). In the case of DSB within the nucleolus, ATM allows the blocking of polymerase I and its release of the nucleolus (94). ATM could conceptually also inhibit the transcription of some viral genes, such as the main EBV oncogene LMP1. ATM deficiency in AT patients could therefore release this inhibition, contributing to lymphomagenesis (Figure 2C). ATM could also participate in the restriction of EBV latency by promoting the transition from type III to type II latency and/or from type II to type I latency. The large number of EBV-associated Hodgkin's lymphomas, described as being derived from type II-latency-infected B-cells (95), suggests that the restriction of latency may not efficiently occur in AT patients.

Humanized mice are a potent model to study the early stages of EBV infection, establishment of latency III, and the immune system response (96). However, B-cell ontology is not complete in these mice, with little germinal center reactions or BCR maturation, impeding the study of latency II and I. Moreover, lytic infection cannot take place because of the absence of human epithelial cells. Recent advances have greatly improved the ontogeny of B-cells in these mice (97) and could open a new field for EBV study. An infection of these cells by EBV has, to our knowledge, not been yet assessed.

Studies on the mechanisms of EBV-induced lymphomagenesis in AT patients may shed light on the pathways involved in the control of chronic EBV infection. This will have a significant impact on the understanding of the physiopathology of EBV-LPD, even outside of the context of AT. The tumor suppressor role of ATM is highlighted by the frequent somatic mutations of *ATM* in many lymphoid malignancies. This understanding could allow the exploration of new therapeutic targets in these lymphomas, for which there is still no effective treatment targeting EBV, and in patients with AT where the usual therapeutic approaches by cytotoxic agents are limited because of their toxicity in the context of DNA repair abnormalities.

## Author Contributions

MT performed the research and wrote the manuscript. OH wrote the manuscript. FS supervised the research and wrote the manuscript.

### Conflict of Interest Statement

The authors declare that the research was conducted in the absence of any commercial or financial relationships that could be construed as a potential conflict of interest.
